# Coconut Oil as a Novel Approach to Managing Radiation-Induced Xerostomia: A Primary Feasibility Study

**DOI:** 10.1155/2020/8537643

**Published:** 2020-08-06

**Authors:** Alexandra E. Quimby, Debora Hogan, Diana Khalil, Matthew Hearn, Colette Nault, Stephanie Johnson-Obaseki

**Affiliations:** ^1^The University of Ottawa, Department of Otolaryngology—Head & Neck Surgery, Ottawa, Ontario, Canada; ^2^The Ottawa Hospital, Ottawa, Ontario, Canada; ^3^Chatham-Kent Health Alliance, Chatham, Ontario, Canada

## Abstract

**Background:**

Xerostomia is a common complication following radiation therapy for head and neck cancer (HNC), for which there is no single, universally accepted therapy. Coconut oil has been anecdotally suggested to provide relief for this complication. This study sought to examine the feasibility and effectiveness of coconut oil as a therapy for radiation-induced xerostomia.

**Methods:**

A feasibility study was performed among 30 patients with xerostomia subsequent to radiation for HNC. Coconut oil samples were provided along with a protocol for use over a 2-week period and the option to continue if they found it beneficial. Patients were also instructed to keep diaries to document their patterns of use. The Xerostomia-related Quality of Life Scale (XeQOLS) was administered at baseline and 3-month follow-up. Descriptive methods were used to summarize patterns of coconut oil use and paired *t*-tests were used to assess changes in XeQOLS scores over time.

**Results:**

The mean total duration of coconut oil use during the study period was 16 days (1–71). The average number of uses per day was 3 (1–5), with an average amount per use of 5 mL (1.2–8.5). Twelve patients (41.4%) continued coconut oil use beyond the advised period. There was no statistically significant difference in XeQOLS scores pre- and post-treatment. There were no adverse events during the study period.

**Conclusions:**

The use of coconut oil as a treatment strategy for xerostomia post-HNC radiation is feasible, inexpensive, and safe. This study demonstrates that there may be a group of HNC patients that benefit from its use.

## 1. Introduction

Xerostomia (dry mouth) is a common complication in patients treated with radiotherapy for head and neck cancer (HNC), with 60–100% of patients experiencing some degree of xerostomia [1–4]. Radiation, either alone or as an adjuvant to primary surgery, is a mainstay in the treatment of HNC. With cancer of the head and neck being the sixth most commonly diagnosed cancer worldwide, the burden of xerostomia on both patients' quality of life and the healthcare system should not be underestimated [4, 5]. The incidence and intensity of xerostomia as a side effect are proportional to both the dosage of radiation used and the amount of salivary gland tissue included in the radiation field [6]. The implications of xerostomia are wide-ranging and can include difficulty chewing and swallowing, impaired phonation, altered taste sensation, dental carries, oropharyngeal candidiasis, systemic malnutrition, and weight loss. Several studies have demonstrated a significant negative impact of xerostomia on patients' quality of life [7, 8, 9, 10].

There is no standard, universally effective therapy for the treatment of radiation-induced xerostomia. Salivary gland transplant and intensity-modulated radiotherapy are two approaches to preventing radiation-induced xerostomia, though neither is 100% effective at preventing the complication, and neither has been universally adopted [11–15]. Most other treatments are directed at alleviating symptoms and are nonspecific in nature. Examples include lifestyle modifications (discontinuation of smoking, dairy, and other dietary products that thicken saliva), artificial saliva preparations (e.g., Biotene [16]), and parasympathetic agents that stimulate saliva production (e.g., pilocarpine). Unfortunately, these treatments have limited efficacy, can be costly, and medical treatments such as pilocarpine can be associated with significant side-effects [17–19]. Other therapies such as acupuncture and hyperbaric oxygen have been studied but lack convincing evidence of efficacy [20, 21].

Coconut oil, a natural food product commonly used as an additive in cooking as well as in various cosmetic preparations, has been anecdotally suggested to ameliorate the symptoms of xerostomia following radiation treatment for HNC. The mechanism of xerostomia relief from coconut oil may be related to its ability to “coat” the mouth, forming a barrier to keep mucosal surfaces moist. Attestations of the benefits of coconut oil in this setting have come from both patients and allied health care professionals involved in the care of HNC patients post-radiation at our institution. Coconut oil has never been formally studied as a strategy for managing radiation-induced xerostomia.

This study sought to investigate, first, the feasibility of using coconut oil as a management strategy for radiation-induced xerostomia in patients treated for HNC, and second, whether coconut oil could provide relief of xerostomia in this patient population.

## 2. Methods

### 2.1. Study Design and Objectives

This study was designed as a pilot case series to assess, primarily, the feasibility of using coconut oil as a management strategy for radiation-induced xerostomia. As a secondary outcome, we sought to assess the efficacy of coconut oil in alleviating symptoms of xerostomia.

### 2.2. Study Population

Our study was conducted at the Ottawa Hospital Cancer Centre (TOHCC), Ottawa, ON, Canada. Patients who had completed treatment for head and neck cancer at least 18 months prior, received external beam radiotherapy at a dose of at least 50 Gy to the head and neck, and subsequently subjectively experienced some degree of xerostomia, were our study population of interest. To be included in the study, patients were required to be proficient in English, be competent to consent to study inclusion, and be willing to comply with the study protocol and follow-up schedule. Any known allergy or sensitivity to coconut oil was considered an exclusion criterion. Xerostomia was operationally defined as the subjective experience of dry mouth following completion of radiation treatment.

### 2.3. Study Protocol

HNC patients who met inclusion criteria were identified at follow-up appointments at an outpatient clinic at TOHCC. Those who had previously provided consent to be approached for research purposes were flagged based on provider clinic lists and electronic medical records (EMRs), and approached by the Clinical Research Coordinator who obtained informed consent for patients who chose to participate. A sample size of 30 patients was selected out of convenience to conduct this pilot study.

Following obtaining written consent for study participation, participants were given a standardized sample of coconut oil in kind and provided with instructions on the use of coconut oil for study purposes. A suggested regimen (based on anecdotal patient reports and consensus among the clinical care team) of coconut oil use prior to meals and at bedtime, with the individual coating his or her mouth with the product at each application, was provided. However, participants were unrestricted in terms of the use of the coconut oil and were invited to titrate its frequency and quantity of use in a way that best alleviated their symptoms of xerostomia. Participants were asked to use coconut oil for a two-week period and given the option to continue use beyond this initial period if they found benefit from the oil. The sample of coconut oil provided to patients was sufficient to last a two-week period of use. Beyond the initial provision of coconut oil and instructions for its use, patients were not prompted in any way regarding continued coconut oil use throughout the study period.

A follow-up visit was scheduled approximately three months following the baseline study visit and marked study completion for each participant. This duration was selected to coincide with the routine interval of follow-up visits for HNC patients following treatment.

Participants were informed that if at any time during the two-week period, they found the use of coconut oil either intolerable or inefficacious, they could revert to their prior method of alleviating their xerostomia.

### 2.4. Outcome Measurement

Participants were asked to keep a diary chronicling their patterns of coconut oil use during the two-week study period. This diary was provided to patients at the time of their baseline study visit and returned to the Clinical Research Coordinator at the study follow-up visit.

At the baseline visit, prior to commencement of the study period, patients were asked to rate their symptoms of xerostomia using a previously validated 15-question xerostomia-related quality of life scale (XeQOLS). The XeQOLS includes questions pertaining to each of four major domains which must be considered in determining the health-related quality of life: physical functioning, personal/psychological functioning, social functioning, and pain/discomfort [16, 22, 23]. Patients then completed the XeQOLS again at the time of the study follow-up visit.

Data on baseline demographic variables of interest were also collected, including age, sex, primary cancer site, dates of surgery, start and end dates of radiation, total dose and side-effects of radiation, past medical history, and medications. Patients' past medical history was used to calculate their Charlson comorbidity score [24]. Lists of medications provided were assessed for any potentially anticholinergic medications.

Outcomes of interest were as follows:

1. The percentage of participants who continued coconut oil use beyond the 2-week trial period
2. Parameters (frequency, quantity, and timing) of coconut oil use followed by participants
3. Changes in XeQOLS scores over the three-month study period

### 2.5. Statistical Analysis

Descriptive methods were used to summarize patient demographics and patterns of coconut oil usage over the study period. Paired *t*-tests were used to assess changes in XeQOLS scores over time. A linear regression model was used to assess for any association between patterns of coconut oil use (total days used, frequency of use, amount of oil per usage, and total amount per day) and changes in XeQOLS scores over the study period. Logistic regression was used to assess for associations between baseline variables and continued use of coconut oil; and between baseline variables and improvement in XeQOLS scores. A two-sample *t*-test assuming unequal variance was used to assess for an association between baseline XeQOLS score and anticholinergic medication use. Pearson's correlation coefficients (*r*) were used to assess for associations between baseline XeQOLS scores and other baseline demographic variables including total radiation dosage, time since radiation completion, and Charlson comorbidity scores. Negative values of *r* represented an inverse association and positive values of *r* represented positive associations; *t*-tests were used to assess whether correlation coefficients differed significantly from *r* = 0.

In analyzing XeQOLS scores, each question was scored on a 5-point scale (0–4), with higher scores corresponding to worse xerostomia-related symptoms, as previously reported [23]. Average scores were calculated for each of the four domains assessed within the XeQOLS: physical functioning (questions 1, 6, 10, 12), personal/psychological functioning (questions 8, 13, 14, 15) social functioning (questions 4, 5, 11), and pain/discomfort (questions 2, 3, 7, 9). An overall score was calculated by taking the average of the mean scores in each of these four domains. The range of possible overall scores on the XeQOLS is therefore 0–4.

Cronbach's alpha was used to calculate interitem correlations on the XeQOLS among study participants.

A *p*-value of 0.05 was used as the cut-off for statistical significance. Statistics were performed using STATA software, version 15 (StataCorp, College Station, TX).

### 2.6. Safety and IRB Approval

Coconut oil is approved by Health Canada as an oral coating agent, defined as “substances used to coat a solid formulation in order to aid in stability or improve the taste or odour.” [25]. It is approved by the FDA as a food additive [26]. A recent review in the Journal of Nutrition concluded that dietary saturated fatty acids as found in coconut oil do not have any appreciable systemic inflammatory or cholesterol-raising effects [27]. There is no data to suggest appreciable adverse effects of coconut oil as a lubricant. The main potential detriment associated with the use of coconut oil which we could foresee was weight gain as a result of the ingestion of the substance, given its high lipid content. However, it should be noted that many study participants were cachexic and already using other methods of supplementation for weight gain at the time of study inception. Patients were forewarned of this possible side effect of the treatment and provided with the nutritional information as per the manufacturer's label. Participant diaries were formatted to capture any adverse events.

Our study was approved by our hospital REB (Ottawa Health Science Network Research Ethics Board [OHSN-REB]) (protocol no. 20170139-01H). The trial was registered with ClinicalTrials.gov (NCT identifier 03176368).

## 3. Results

Between October 5, 2017, and January 25, 2018, 50 patients were prescreened for eligibility at outpatient follow-up appointments and invited to participate in the study. Of these, 10 patients were excluded due to previous use of coconut oil for xerostomia, and 10 declined to participate. Of the 30 participants recruited into the study, complete follow-up information (completed diaries and XeQOLS questionnaires) was available for 26 participants. Demographics of enrolled participants are shown in Table 1.

There was no significant association between baseline anticholinergic medication use and baseline XeQOLS scores (*t* = −1.7110, *p* = 0.12). There was no significant association between baseline XeQOLS scores and total radiation dosage, time since radiation, or Charlson comorbidity score.

Completed diaries were available for 29 participants. 19 patients (73.1%) used coconut oil for the duration of the two-week study period. The mean total duration of coconut oil use during our study period was 16 days (range, 1–71 days; IQR 2–30 days). The average number of uses per day was 3 (range, 1–5 uses per day; IQR 2–4 uses per day). The average amount of coconut oil per use was 5 mL (range, 1.2–8.5 mL; IQR 2.5–7.5 mL), and the average total amount of coconut oil used per day was 16 mL (range, 5–30 mL; IQR 10–25 mL). The most common triggers for use were mealtimes and before sleep (100% of patients). Twelve participants (41.4%) continued using coconut oil beyond the advised two-week period. Of participants who continued coconut oil usage the mean number of uses per day was 4 (range, 1.6–5 uses per day; IQR 4 uses per day); the average amount of coconut oil per usage was 5 mL (range 2.5–8.5 mL; IQR 5–5.2 mL); and the average total amount of coconut oil used per day was 20 mL (range 10–26.4 mL; IQR 15.75–20 mL). These participants who continued to use coconut oil did not necessarily use the product every day.  Table 2 summaries parameters of coconut oil use in our study population.

There was no significant association between any baseline demographic variable (age, sex, Charlson comorbidity score, use of anticholinergic medications, primary tumor site, radiation dosage, and time since radiation), and the odds of continued coconut oil use beyond the initial two-week period.

**Figure 1:**
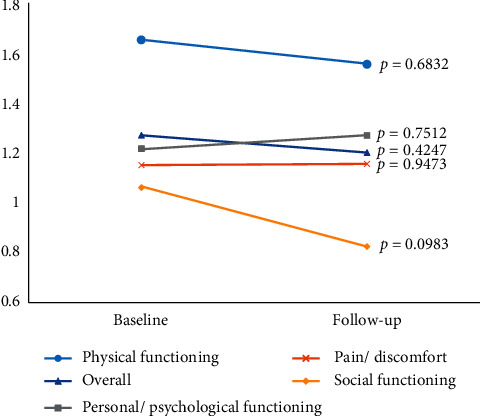
Changes in mean XeQOLS scores from baseline to follow-up.

Completed XeQOLS questionnaires from both baseline and follow-up visits were available for 26 participants. The mean baseline XeQOLS score was 1.28 (range 0.06–2.54; IQR 0.5–2.06). The mean follow-up XeQOLS score was 1.20 (range 0.25–2.29; IQR 0.5–2.08). There was no significant difference in XeQOLS scores pre- and post-treatment, both among the entire study group (*n* = 26) (*t* = 0.8115, *p* = 0.42) and among those participants who continued coconut oil beyond the study period (*n* = 12) (*t* = 1.0454, *p* = 0.32) (Figure 1). There was also no significant difference in scores pre- and post-treatment among any individual domain of the XeQOLS (physical functioning, personal/psychological functioning, social functioning, and pain/discomfort).

Among the entire study group, 13 participants (50%) reported post-treatment XeQOLS scores which were significantly increased from pre-treatment scores (*t* = -8.30, *p* < 0.0001). Seven of the participants who reported worse XeQOLS scores at follow-up continued coconut oil use beyond the trial period. Among the group of patients (*n* = 13) with worse XeQOLS scores at follow-up, analysis of individual domains within the XeQOLS demonstrated significantly worse scores within the personal/psychological functioning domain (*t* = −2.41, *p* = 0.03) and the pain/discomfort domain (*t* = −3.15, *p* = 0.01). Among the 5 participants who continued coconut oil use beyond the recommended period and whose XeQOLS scores decreased (improved) post-treatment, the decrease was significant (*t* = −3.39, *p* = 0.027).

There was no significant association between parameters of coconut oil usage (number of days used, number of uses per day, amount per usage, and total amount per day) and changes in XeQOLS scores.

There was no significant association between any baseline demographic variable and the odds of improvement in the XeQOLS score.

There was a good interitem correlation among questions on the XeQOLS at both baseline and follow-up visits, as indicated by Cronbach's alpha scores of 0.8417 and 0.8279, respectively (where alpha >0.7 indicates high internal consistency).

There were no adverse reactions to coconut oil use reported during the study.

## 4. Discussion

The overall health- and quality-of-life-related effects of xerostomia should not be underestimated. With the incidence of xerostomia reaching 100% in some series of irradiated head and neck cancer patients, it is clear that this problem is exceedingly pervasive [2]. Operative salivary gland transplant can be technically demanding and is not routinely performed at all centres where head and neck cancer patients are treated, and intensity-modulated radiation therapy is not wholly effective in preventing xerostomia. Currently available treatments for xerostomia are similarly variably effective at best.

Coconut oil is an attractive low-cost, low-side effect, and feasible option for the management of xerostomia in irradiated head and neck cancer patients. It is a natural product which offers ease of use and anecdotally has been suggested to ameliorate some patients' xerostomia.

In our pilot case series, we found that almost half (41.4%) of participants found benefit in the use of coconut oil as evidenced by continued use beyond our recommended trial period. This finding demonstrates participant motivation to continue the use of the product since they were required to purchase their own coconut oil to maintain use beyond the initial two-week period. We were able to delineate parameters of coconut oil application (including amount, frequency, and timing) that were found to be useful by head and neck cancer patients. No participant used the product every day from baseline to follow-up (approximately 90 days), as indicated by a maximal duration of use of 71 days during the study period. This indicates that some patients may find the product helpful on an as-needed basis, with a frequency of use less than daily.

We did not detect any clinical variable that was able to predict ongoing coconut oil use (i.e., to suggest particular patients who might benefit from its use). However, our inability to detect such associations may be the result of our small sample size.

Interestingly, despite a large proportion of our study participants continuing coconut oil use beyond the trial period, there was no overall difference in XeQOLS scores pre- and post-treatment, both among the entire group of study participants who completed both surveys (*n* = 26) and among those who continued coconut oil use (*n* = 12). Further analysis of pre- and posttreatment XeQOLS scores revealed that among the entire study group, 13 (50%) of patients demonstrated significantly increased (worse) XeQOLS scores at follow-up (post-treatment) compared to baseline (pre-treatment) (*p* < 0.0001). Similarly, among participants who continued oil use, only 5 reported decreased XeQOLS scores at follow-up, with the other 7 reporting paradoxically increased overall XeQOLS scores. Among these patients who experienced worse XeQOLS scores post-treatment, analysis of specific domains within the XeQOLS demonstrated significantly worse scores specifically within the pain/discomfort and personal/psychological functioning domains. Questions on the XeQOLS related to pain/discomfort included “my mouth/throat dryness causes discomfort,” “my mouth/throat dryness causes a lot of worry or concern,” “my mouth/throat dryness makes me nervous,” and “my mouth/throat dryness keeps me from enjoying life.” Questions related to personal/psychological functioning included “my mouth/throat dryness makes me concerned about the looks of my teeth and mouth,” “my mouth/throat dryness reduces my general happiness with life,” “my mouth/throat dryness affects all aspect of my life” and “if you were to spend the rest of your life with your mouth/throat dryness just the way it is now, how would you feel about this?”. It is possible that the significant increase in XeQOLS scores within the pain/discomfort and personal/psychological functioning domains was due to participants' becoming more aware of their xerostomia as a result of study participation. Further investigation of this theory could be completed by way of qualitative research techniques to explore the lived-experience of participants, but this was not within the scope of the present study.

The ability to draw conclusions regarding the efficacy of coconut oil in improving xerostomia among head and neck cancer patients is limited by the small sample size of this study. However, in keeping with the primary aim of the study, we have been successful in assessing the feasibility of coconut oil as a therapy for radiation-induced xerostomia in this patient population. Furthermore, we have been able to outline parameters of coconut oil use that were found to be effective by study participants. This information would be useful in guiding future research, including head-to-head comparison of coconut oil and other commercially available products (e.g., Biotene) used in the treatment of xerostomia.

## 5. Conclusion

Xerostomia is a pervasive problem following irradiation for head and neck cancer, with wide-ranging effects on patients' overall health and quality of life. Coconut oil offers a feasible, inexpensive, and safe alternative to the currently available, imperfect therapies for xerostomia. This pilot case series has demonstrated that there may be a population of head and neck cancer patients who benefit from the use of coconut oil. It has served to outline parameters of coconut oil use which may be of benefit, and which could guide further study into the efficacy of coconut oil as a therapy for radiation-induced xerostomia.

## Figures and Tables

**Table 1:** Cohort baseline demographic variables.

Variable	Category	Value
Age (years)	Mean (SD)	67 (8)
Gender	Male	20 (66.67%)
	Female	10 (33.33%)
Primary HNC site	Oral cavity	14 (46.67%)
	Oropharynx	6 (20%)
	Nasopharynx	3 (10%)
	Larynx	1 (3.33%)
	Hypopharynx	1 (3.33%)
	Unknown primary	5 (16.67%)
Radiation dosage (Gy)	Mean (SD)	65.8 (6.6)
Time since completion of radiation (months)	Mean (SD)	36 (18)
Charlson comorbidity score	Mean (SD)	2.8 (1.3)
Baseline anticholinergic medication use	Yes	8 (26.67%)
	No	22 (73.33%)

HNC = Head & Neck Cancer

**Table 2:** Coconut oil use parameters.

Variable	Measure	Value
Total duration of use, days	Mean (IQR)	16 (2–30)
Uses per day	Mean (IQR)	3 (2–4)
Amount per use, mL	Mean (IQR)	5 (2.5–7.5)
Amount per day, mL	Mean (SD)	16 (10–25)
Triggers:	Percent	
Mealtimes (%)		93%
Bedtime		67%

IQR = interquartile range.

## Data Availability

The data used to support the findings of this study are available from the corresponding author upon request.
